# Principal spatiotemporal mismatch and electricity price patterns in a highly decarbonized networked European power system

**DOI:** 10.1016/j.isci.2022.104380

**Published:** 2022-05-10

**Authors:** Leon Joachim Schwenk-Nebbe, Jonas Emil Vind, August Jensen Backhaus, Marta Victoria, Martin Greiner

**Affiliations:** 1 Department of Mechanical and Production Engineering, iClimate, Aarhus University, Inge Lehmanns Gade 10, 8000 Aarhus C, Denmark; 2 Centrica Energy Trading A/S, Ørestads Boulevard 73, 9th Floor, 2300 København S, Denmark

**Keywords:** Energy resources, Energy policy, Energy management, Energy Modelling

## Abstract

As the European power system decarbonizes, the variability of the mismatch between renewable generation and demand, as well as that of electricity prices, are expected to increase substantially. Because mismatch and prices show complex temporal and spatial interaction, we propose the use of principal component analysis (PCA) to investigate them. We unveil their main spatiotemporal patterns, examine their cross-correlation, and their dependence on the transmission capacity expansion and ${\text{CO}}_{2}$ emissions reduction in a highly renewable cost-optimal electricity model. We find that the majority of variance in both the mismatch and price time series is explained by just three principal components (PCs). Hence, a convenient switch of basis vectors allows expressing the time series as combinations of few components which are shown to have intuitively interpretable structures. Moreover, we find that the temporal coherence between the first three PCs of mismatch and prices are substantially reinforced as the system decarbonizes.

## Highlights

• Modeling the future European energy system with a high share of renewable resources
• Variability of mismatch between renewable generation and demand and prices increases
• PCA method proven potent to unveil their characteristic spatiotemporal patterns
• Few PCs suffice to represent their variability, enabling various applications

## Graphical abstract

**Figure:**
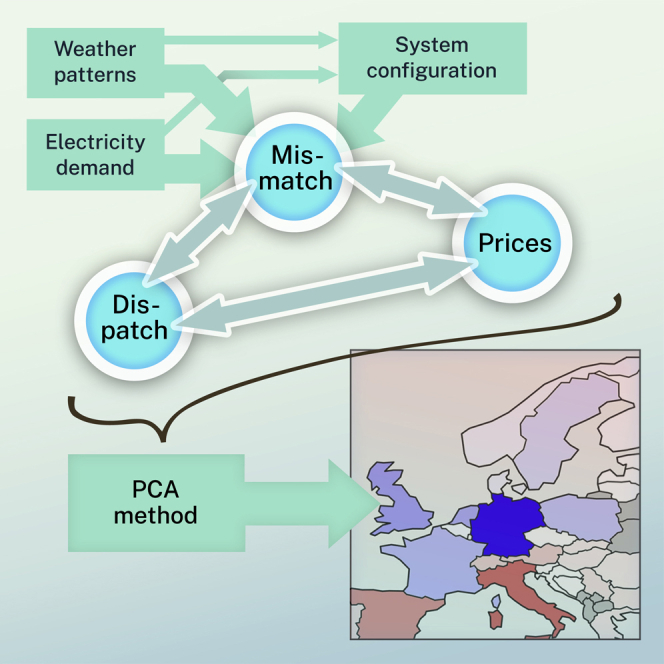

## Introduction

The European power system is rapidly decarbonizing, and the future system is likely to be based primarily on renewable generation. Consequently, the appearance of periods of high and low renewable generation increases in importance and might challenge the grid. The variable renewable technologies solar photovoltaics (PV), onshore wind, and offshore wind are good candidates for constituting the cornerstone of a cost-optimized fully decarbonized European energy system. Their massive deployment enables short-term climate action, which is shown to pay off in the long run (Victoria et al., 2020). However, a future strongly interconnected European energy system with high solar and wind penetration entails additional challenges such as increased variability of the mismatch between renewable generation and demand and of the electricity prices. We denote as mismatch the energy imbalance between the variable renewable generation from wind, solar, and run-of-river and the electricity load. Hence, the mismatch is also known as the negative net load.

Modeling energy systems require powerful optimization frameworks. In order to accurately capture the interactions of intermittent renewable generation, storage operation, and the power flows in the network, the models need to be spatiotemporally finely resolved and entail enough data to capture the diurnal, weekly, and seasonal effects of the weather. A state-of-the-art optimization framework specifically developed for this purpose is the open-source package PyPSA (Brown et al., 2018a). Similar models include Calliope (Pfenninger and Pickering, 2018), LUT-Energy model (Bogdanov et al., 2019), and many others (openMod, 2022). Typically, models perform a combined infrastructure investment and dispatch optimization with optimal power flow calculations. The objective of the optimization is to minimize the total annualized cost of the energy system. Several papers have used PyPSA-based models to show the benefits of increased interconnection capacity between the European countries in a highly renewable electricity system (Schlachtberger et al., 2017), assess how policy constraints and cost parameters influence the results (Schlachtberger et al., 2018), evaluate the special role of storage technologies throughout the decarbonization pathway (Victoria et al., 2019), and determine the influence of CO_2_ emission quotas in the European electricity system of the near future (Schwenk-Nebbe et al., 2021).

While most studies so far have focused on the configuration of the resulting systems, we now focus on the interplay between the weather-driven renewable power generation, the dispatch of the system, and the electricity prices. On the one hand, the mismatch between renewable generation and the electrical load depends on the weather patterns and electricity demand, which are both exogenous to the model, as well as on the resulting optimized capacity layout and dispatch decisions endogenously determined. On the other hand, the electricity prices are mainly an output from the model and are very dependent on the constraints on the system, like the exogenous decarbonization goal. We investigate first intracorrelations between different types of renewable generation and the load, intracorrelations between the different response terms from the system, and intercorrelations between the previous elements. For these, we analyze their dependence on the transmission expansion and ${\text{CO}}_{2}$ emission reductions. Subsequently, we decompose the mismatch and price time series to investigate their dominant spatiotemporal patterns. The emerging patterns are projected back onto their respective contributions.

Because the mismatch and electricity price variables fluctuate in time and space, we use principal component analysis (PCA) to unveil the dominant patterns. Previously, a PCA was applied to a highly simplified networked model of a highly renewable European electricity system (Raunbak et al., 2017). In that analysis, the system configuration was based on heuristic rules and not subject to optimization. The authors showed that a small number of principal components (PCs) could entail most of the variability patterns and justify most of the necessary backup and transmission capacity of the system.

Several studies have investigated the electricity pricing dynamics of decarboniszd grids. Strong decarbonizations lead to large deployment of wind and solar generation. Their zero marginal cost changes the price patterns fundamentally. Tightening the ${\text{CO}}_{2}$ emission quota leads to a severe increase in occurrences of very low electricity prices in (Mallapragada et al., 2021) while also more periods of high prices are observed. The spatiotemporal dynamics of highly decarbonized electricity systems is analyzed for the US grid in the study by Brown and Botterud (Brown and Botterud, 2021). With large shares of renewables already on the grid in several regions, one observes the merit order effect of renewable energy systems. Several studies confirm these effects, like the study by Kolb et al. (Kolb et al., 2020) shows the effects of large wind and solar generation on the electricity prices in Germany. To accelerate the renewable transition, carbon taxes are introduced and tightened in several markets. They have significant implications for market behavior and thereby electricity prices (Wong and Zhang, 2022). Furthermore, they also couple energy markets beyond the direct cross-border electricity flows through correlations between carbon and electricity prices (Moutinho et al., 2022) and allowance swap trading between emission markets (Kanamura, 2016). In recent years, issues of stranded assets and a just transition are lifted into the spot light. These are also expected to influence the electricity prices as investors need to price in the risk of asset stranding (Sen and von Schickfus, 2020). Socio-economic parameters are important both in the greater perspective of economically vulnerable countries (Wang and Lo, 2021), but also the need for electricity to be affordable by vulnerable consumers (Voicu-Dorobanțu et al., 2021). For power system analysis, PCA has previously been applied to investigate electrical consumption patterns in buildings in (Burgas et al., 2014). Similarly, PCA has been applied to predicting the reliability of transmission grids (Zheng et al., 2013) and to build forecast models of short-term electricity prices (Maciejowska et al., 2020).

The main novelty of this publication is that we go beyond optimizing a highly decarbonized European electricity system and quantify the relations between the weather-driven mismatch on the one hand and the electricity prices on the other hand. A PCA is applied to unveil the main spatiotemporal patterns of the mismatch and electricity prices, and to investigate the correlation between them. The mismatch depends on input variables but also optimization decisions like the chosen system configuration, while the nodal electricity prices are output variables obtained via the Lagrange multipliers from the nodal balancing constraint on the model (see Equation 1).

The research questions we want to answer in the current study can be formulated as follows. In a power system with high renewable penetration, how does the fluctuating weather-driven mismatch drive the dispatch decisions? And subsequently, what influence does the dispatch have on the electricity prices? Moreover, to what extent are the prices driven by the mismatch dynamics? How do the average prices and their variances depend on the strength of the transmission grid and the level of decarbonization? We set out to find the principal spatiotemporal patterns across Europe describing these interactions. Hence, we investigate how the dominant weather-driven mismatch patterns contribute to the dispatch decisions of gas-fueled backup generation, hydro reservoir units, imports and exports, and the prices. Furthermore, we map out by how much the dominant mismatch patterns are generated by wind or solar generation or by the inelastic electricity demand.

## Results

We begin by introducing the base case scenario. This scenario represents a highly decarbonized future European electricity system heavily based on wind and solar generation. In Mismatch variance, we inspect the variance in the mismatch time series. We introduce parametrized scenarios around the base case scenario. These scenarios will later be investigated in further detail to explore the influence of the transmission grid strength and varied degree of decarbonization of the electricity system. In Mismatch principal components, we dive deeper into the base case scenario in order to investigate the emerging spatiotemporal patterns in the European mismatch. We observe the emergence of a few strong spatial patterns, called principal components (PCs), which explain most of the total mismatch variance.

The applied model assumes perfect competition, market equilibrium, and perfect foresight. The model is a greenfield simulation but fixes reservoir hydro, pumped hydro storage, and run-of-river to existing capacities in 2020 as potentials are assumed to be fully exploited. Cost assumptions of components are based on cost projections for the year 2030 and a discount rate of 7% is applied. We assume 2030 costs projection to account for the expected cost reduction in most technologies while not using a far-future year for cost assumptions that will imply large uncertainty.

As the transition toward carbon neutrality requires fast decarbonization of the electricity sector, we define our base case scenario based on the requirement to reduce its emissions by 95% compared to electricity-generation-related emissions in 1990. Our base case scenario will form the basis of our analysis of a future electricity system in Europe. In the base case scenario, we allow the optimization to extend the cross-border transmission lines up to a total transmission volume (line capacities times line lengths) equal to twice the transmission volume of today’s system.

**Figure 1:**
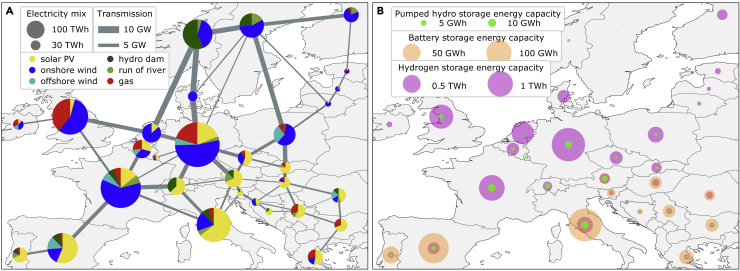
Configuration of the base case scenario, which enforces a 95% ${\text{CO}}_{2}$ emission reduction compared to 1990 and has twice the cross-border transmission capacity of today (A) shows the annual electricity mix composition. We see the contributions from the variable renewable generators solar PV, on- and offshore wind, run-of-river, the dispatchable generation from hydropower dam reservoirs, and the backup generation from gas-fueled installations. Energy can be stored chemically in batteries, as potential energy in pumped hydro storages (exogenous to the optimization), and converted to hydrogen and back. (B) shows a plot of the installed energy capacity of the storages. Note the different scales of the discs on (B) for the different technologies.

The cost-optimal solution found by the solver, given the constraints of the base case scenario, leads to a system that is visualized in Figure 1. On (A), we depict the electricity mix consisting of different electricity supply sources together with the strength of the transmission grid. (B) supplements this view with the installed storage energy capacities. The obtained European generation mix is dominated by onshore wind and solar PV generation. Large amounts of hydropower (exogenous to the system) are found in the north, and solar generation tends to increase with decreasing latitude. Large hydrogen storage capacities and reinforced transmission lines coincide with the wind-dominated northern countries. This has previously been observed in the study by Victoria et al. (Victoria et al., 2019). Similarly, battery storage is found primarily in countries with extensive solar power generation.

### Mismatch variance

**Figure 2:**
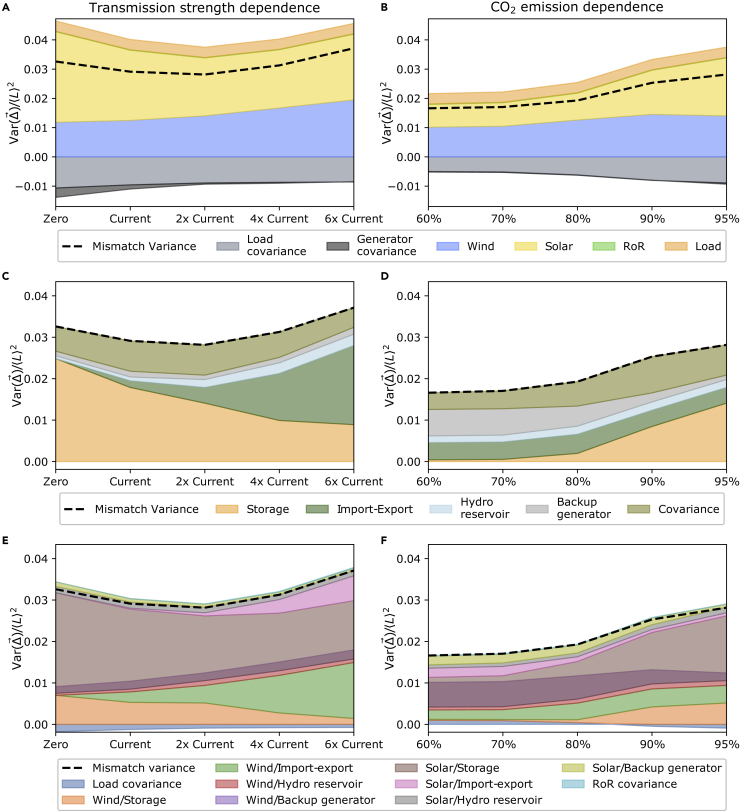
Variance of the mismatch between the variable renewable generation and the electricity demand for three decompositions in various scenarios (A) shows the contributions by the renewable generators, their combined covariance terms, the load, and the combined load covariance terms as a function of the transmission grid extension. (B) Similarly, (B) shows the same mismatch variance contributions as a function of the ${\text{CO}}_{2}$ emission reduction. (C and D) show the system response to the mismatch in terms of the storage, import and export, hydro dam reservoir, fossil-based backup capacity, and the combined covariance term contributions for the transmission expansion and emission reduction, respectively. Finally, (E) and (F) show the cross-term contributions to the mismatch variance between the terms from the first and second row panels. All cross-term covariances, including the load, are combined into a single contribution in the figure and the same is done for the run-of-river (RoR) contributions as they are both small. The thick dashed lines represent the total mismatch variance in all panels, and they are always the sum of their shown contributions (where some covariances are negative). The total mismatch variance is naturally the same in the three left and right panels, respectively. Note that the center scenario in (A), (C), and (E) corresponds to the rightmost scenario in (B), (D), and (F) as they both have twice the current total grid strength while being highly decarbonized. See also Figure S1.

Before introducing the main results from the PCA, we present the mismatch variance in the different investigated scenarios that are parametrized by transmission strength and total annual system emission. This is done by imposing a constraint on the system that sets an upper bound to the total transmission capacity and the total ${\text{CO}}_{2}$ emissions, respectively. We define parametrized scenarios that sweep across the transmission and ${\text{CO}}_{2}$ constraints to investigate the system operation under variable parametrized scenarios. To explore the overall characteristics of the variance in the mismatch time series, we turn to the three ways of expressing the mismatch between the renewable generation and the load as defined in the Equations 3, 5, and 6. A visual representation of the results is given in Figure 2. Through the variable renewable generation, the variance of the mismatch is highly weather-dependent. Let us first look at the variable renewable generation and load contribution to the variance as shown for the transmission expansion dependence in (A) and for the emission reduction parametrization in (B). The mismatch variance is dominated by its solar and wind contributions. In (A), we see that a transmission expansion leads to an increase of the relative importance of the wind contribution but the solar contribution remains the strongest. A stronger transmission infrastructure enables wind generation to be balanced over longer distances smoothing out the weather-driven spatial wind patterns (Victoria et al., 2019). Therefore, the optimization calls for slightly larger capacities that in turn show up as larger contributions to the variance here. The emission reduction dependence in (B) shows that the relative contribution strengths of the individual components remain unchanged.

Let us next turn to the system response contributions shown in the center row of the figure. On (C), we see that larger transmission capacity leads to more significant flows on the links that help smoothen out the weather patterns across Europe over longer distances and thereby, a more significant amount of mismatch variability is explained by imports and exports. Furthermore, on (C), we observe that an increased transmission volume leads to fewer storage needs and in turn, the storage contribution explains less of the mismatch variance. This is a direct consequence of smoothing out intermittent generation over longer distances that leads to less curtailment (as seen in Figure S1) and a decreased need for storage. As can be seen in (D), the need for storage and its contribution to the total mismatch covariance increases for stricter ${\text{CO}}_{2}$ emission reductions.

Lastly, the bottom row in Figure 2 presents the contributions from the cross terms. On the (E) and (F), we also observe that solar correlates with storage use and wind with the import/export injection pattern. (F) shows that reducing emission allowance leads to a more significant solar/storage covariance, as more of the mismatch variance is explained by storage contribution.

### Mismatch principal components

**Figure 3:**
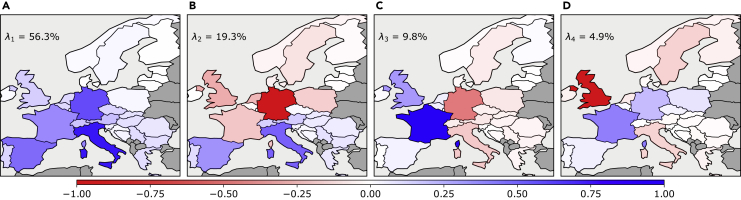
Main PCA projections of the mismatch between renewable generation and demand in the base case scenario (A–D) show a map visualizing the principal mismatch components 1 through 4, respectively. The panels also indicate the associated eigenvalue strengths $\lambda_{k}$. A large part of the variance is explained by the monopole in (A) and dipole in (B) alone. Cumulatively, the first six PCs explain 94.8% of the variance of the mismatch time series.

We start by taking an in-depth look at the characteristics of the time series of the mismatch between renewable generation and demand in the base case scenario. Instead of analyzing each country’s time series, we apply PCA on all data together—mapping out the spatiotemporal patterns on the European level. Figure 3 shows maps of the first four PC eigenvectors with the largest eigenvalue strengths. With seven PCs, we are able to explain more than 95% of the variance in the original mismatch time series. (A) shows a visualization of the first PC eigenvector that has a corresponding eigenvalue strength of 56.3%. Hence, the first PC captures more than half of the mismatch variability. We observe the occurrence of a clear monopole with the same-sign mismatch (recall that the sign of the PC is arbitrary in PCA projections) in all the European countries. Though central and south-west Europe has a larger contribution than the north-eastern part, the strongest emphasis lies on the mismatch in Italy, Germany, Spain, and France. In (B), we see that a dipole pattern in the north-south direction characterizes the second PC. The largest emphasis is on opposite mismatches in Germany and Italy. The corresponding eigenvalue strength is 19.3%. An east-west dipole with a clear emphasis on opposite mismatches in France and Germany describes the third most dominant PC shown in (C). This PC has an eigenvalue strength of 9.8%. The fourth PC visualized in (D) already has a relatively weak eigenvalue strength of 4.9%. The main emphasis is on the United Kingdom. The pattern of the fourth PC could be described loosely as a north-central-south tripole.

**Figure 4:**
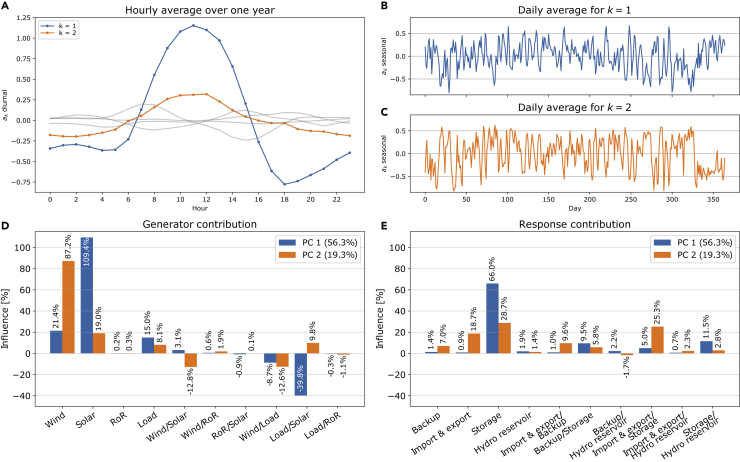
Overview of characteristics of the two first PC amplitudes in the base case scenario for the PCA on the mismatch time series (A) The diurnal characteristics of the two amplitudes are visualized in (A) as the hourly average over one year (together with the next four PCs in the background as a comparison). (B and C) visualize the seasonal behavior of the first two amplitudes, respectively, by showing the daily average values. The following two panels introduce the mismatch PC projections (see the Methodology section). (D) is visualizing the generator and load contribution (13), while (E) shows the response contributions (15) for the first two mismatch PCs.

Let us take a closer look at the time-dependent PC amplitudes. Figure 4 shows various characteristics of the first two amplitudes. The diurnal characteristics of the two amplitudes are visualized in (A). We observe two smaller peaks at the beginning and end of the day, with a distinctive maximum in the middle of the day. As the first PC is a monopole with a positive eigenvector, this coincides with the mid-day production peak of solar PV. The second PC also has a maximum in the middle of the day, even though not as predominant. This corresponds to larger values in the south than in the north in the middle of the day, again coinciding with larger solar PV installations in the south of Europe (see Figure 1). In (B) and (C), we see the daily averages that visualize the annual characteristics of the first and second amplitudes, respectively. The figures do not show a very strong seasonal behavior. For the first amplitude, it is worth mentioning the clear valleys in the early and late parts of the year corresponding to the beginning and end of winter.

Going a step further, we apply mismatch PC projections introduced in Projections. In the bottom two panels of Figure 4, we show the results for the two first PCs. (D) shows the contributions to the mismatch PC from the wind, solar, run-of-river generations and the load, together with their cross-terms. The first PC is clearly solar dominated with a noteworthy contribution from the load/solar interaction. In strong contrast, the second mismatch PC is wind dominated. (E) shows the corresponding response contributions to the PCs. We find that the response to the solar-dominated first PC is clearly based on the storage response. The response to the wind-dominated second PC is more mixed with extensive storage and import/export contributions and contributions from the storage and import/export cross-terms.

### Nodal marginal price principal components

**Figure 5:**
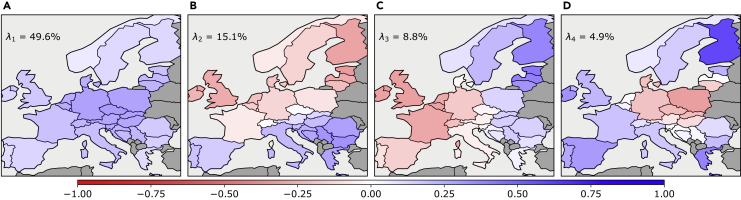
Main PCA projections of the electricity price time series in the base case scenario (A–D) show a map visualizing the first four electricity price PCs, respectively, for the base case scenario. The panels also indicate the associated eigenvalue strengths. The six most dominant PCs describe cumulatively 84.7% of the variance in the electricity prices, and we need 15 PCs to describe 95% of the variance.

Similar to the mismatch, we analyze the nodal marginal price time series of the model in the base case scenario. These represent the hourly electricity prices in the modeled European countries. Figure 5 introduces map visualizations of the four dominant PCs of the nodal electricity prices. Each panel shows one PC and indicates the corresponding eigenvalue strength. We find that the first six PCs of the price PCA explain around 85% of the variance. This is less than for the mismatch PCs. To explain the variance in the prices, we need more PCs than to explain the variance in the mismatch in our modeled European electricity system. This might be caused by the fact that more technologies interplay to determine the electricity price, and since each of them has a capacity layout and optimal dispatch, the resulting spatiotemporal patterns for the electricity prices are more complex. The first PC is visualized in (A). The map shows the eigenvector strengths of the electricity prices. We find a clear monopole, and unlike the first mismatch PC, all countries contribute. The strongest emphasis is on central Europe. That all countries contribute is indicating a stronger coupling between the electricity prices in the individual countries. The first price PC explains 49.6% of the mismatch variability. Turning to (B), we see that the eigenvalue strength is already reduced to 15.1%. We observe a clear dipole structure between the northern and southern parts of Europe. This is again very similar to the corresponding second mismatch PC. (C) shows the emergence of another clear dipole pattern, this time in the east-west direction. The corresponding eigenvalue strength is 8.8%. The fourth PC, visualized in (D), again has a relatively weak eigenvalue strength of 4.9%. As for the mismatch PC, the observed pattern of the fourth price PC can be described loosely as a north-central-south tripole.

**Figure 6:**
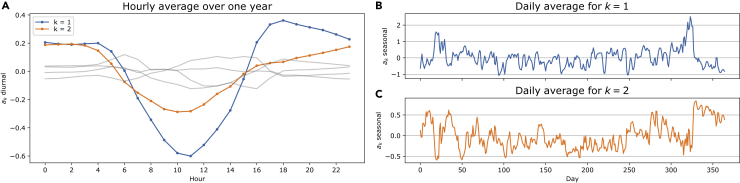
Overview of characteristics of the two first PC amplitudes in the base case scenario for the PCA on the electricity prices (A) In (A), we see the diurnal characteristic of the two first PC amplitudes (with the shape of the next four amplitudes in the background as a comparison). (B and C) visualize the seasonal behavior of the first and second PC amplitude, respectively, by showing the daily average values.

We focus our attention on the first two price PC amplitudes and show several characteristics in Figure 6A which visualizes the diurnal characteristics of the first and second price PC amplitudes. Both show a similar pattern with a strong valley in the middle of the day. Comparing this to the mismatch PC amplitudes, we find that a high average mismatch during the middle of the day corresponds to low average electricity prices in the same mid-day period. Let us then turn to the daily averages shown in the (B) and (C) for the two first price PC amplitudes, respectively. We observe interesting effects in the winter period at the end of the year around day 320 and similarly at the beginning of the year around day 20. For the observations around day 320, the daily average values of the first PC amplitude are much higher than the average. Because this PC describes a monopole and all its eigenvector entries are positive, as seen in Figure 5, this means that we, on average, have higher than normal prices in Europe. (C) in Figure 6 shows that we have an upward spike at the same time, which means that the prices go more up in southern Europe than in northern Europe. Similarly, at day 20, we have high prices in Europe and again higher in southern than northern Europe. If we compare these observations to (B) and (C) in Figure 4, we find that the first PC of the mismatch is lower than average, which in turn means that we have less renewable generation than demand. The low renewable generation leads to the observed increases in electricity prices. In the next section, the correlation of the mismatch and price time series is quantified.

### The coherence of mismatch and price PCs

We have investigated the mismatch patterns and subsequently looked at the spatiotemporal patterns of the electricity prices. In the current section, we want to relate the two by investigating the correlation between the mismatch and price PCs. We apply the coherence measures defined in Coherence measures to shine light on the question of how similar the mismatch and price PC projections are, and to quantify their structural overlap.

The results for the correlation between the first four mismatch and electricity price PCs as measured by the eigenvector coherence (18) are shown in Table 1. We see that there is a strong correlation between the first PCs of the mismatch and prices. The same is observed for the second mismatch PC with the second price PC. Comparing the mismatch PC patterns of Figure 3 with the price PCs in Figure 5, we see that the structural composition of the first two PC maps are relatively similar, explaining the large coherences.

**Table 1:** Eigenvector coherence between the principal components (PCs) of the mismatch and the nodal electricity prices in the base case scenario

Showing the coherence measure as computed by Equation 18. Note that the cell background color intensity corresponds to the cell values for convenient comparison.

The amplitude coherence (19) compares the temporal structure of the mismatch and price PC amplitudes. The amplitude coherence between the four most significant PC amplitudes is visualized in Table 2.

**Table 2:** Amplitude coherence between the PCs of the mismatch and the nodal electricity prices in the base case scenario

Showing the coherence of the PC amplitudes as calculated by the measure defined in Equation 19. Note that the cell background color intensity corresponds to the cell values with blue tones for positive values and red for negative values.

For k=1, we find that the amplitude coherence becomes negative. When the amplitude of the mismatch is positive, then the mismatch is above average. At the same time, it is very likely that the amplitude of the price monopole is negative so that the price is below average. As a result, we find a strong anti-correlation by our measure being negative. A similar argument can be made for the two north-south dipoles of the second mismatch PC with the second price PC. For k=3, we have east-west dipoles for both the PCA of the mismatch and the prices, but with opposite sign, so that the measure becomes positive. Note also that the spatial structure around Germany of the third mismatch PC is no longer similar to the third price PC. The spatial structure of the k>3 PCs is also not comparable and no similar intuitive comparisons can be made.

## Discussions

We return to the mismatch principal components in Transmission grid extension effects on the mismatch for investigating what effects varying the strength of the cross-border transmission grid has. We continue looking at the mismatch in Emission reduction effects on the mismatch where we show the effects of emission reductions on the mismatch PCs. In order to investigate the emerging spatiotemporal patterns in the European electricity prices, we analyze the effects that varying the strength of the cross-border transmission grid and the reduction of ${\text{CO}}_{2}$ emissions have on the price PCs in Effects of transmission grid extension and emission reduction on the prices.

### Transmission grid extension effects on the mismatch

**Figure 7:**
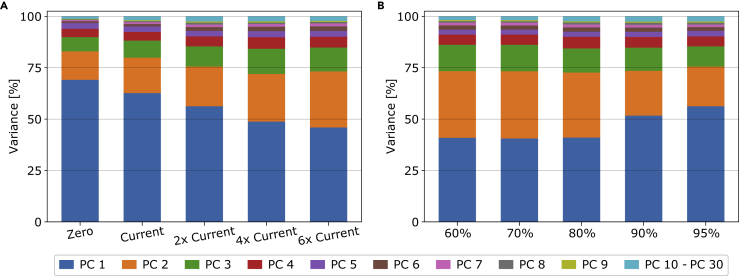
Amount of variance explained by the most dominant mismatch PCs as given by their eigenvalue strengths (A and B) shows the dependence of the PCs on the cross-border transmission expansion constraint. Similarly, (B) shows the mismatch PCs as function of the ${\text{CO}}_{2}$ emission reduction compared to 1990-levels. For each of the scenarios in (A), a 95% ${\text{CO}}_{2}$ emission reduction compared to 1990 values is enforced. In (B), the scenarios have a fixed transmission extension equal to twice the total transmission volume of today. Note that the base case scenario is represented by the center bar in (A) and the rightmost bar in (B).

Let us again deviate from the base case scenario and investigate in further detail the effects that varying grid strengths have on the mismatch time series. The different scenarios have varying amounts of cross-border transmission grid strengths but remain at the same 95% decarbonization relative to 1990 as the base case scenario. We investigate extreme scenarios where we, on the one limit, have no cross-border transmission at all. In a system like this, the countries are fully isolated and need to balance their mismatch internally. On the other extreme, we have a scenario with a strong transmission extension that has a total interconnection capacity of six times the cross-border grid strength we have in Europe today. In between the extreme scenarios, we have grid strengths equal to today’s total transmission, twice, and four times today’s total transmission strength. Let us first turn to (A) in Figure 7 which shows the dependence of eigenvalue strength of the predominant PCs on the transmission expansion. For the leftmost case without any transmission grid, we observe that the first PC captures 69.1% of the variance, whereas the second PC accounts for 13.8%. Increasing the cross-border transmission grid leads to less solar capacity in favor of more wind capacity. The relative importance of the first PC decreases accordingly. With more wind in the system, the wind-dominated PC2 can explain more of the variability in the mismatch. Furthermore, as transmission between the countries increases, the relative importance of PC1 and PC2 becomes more similar. Finally, regardless of the transmission expansion, we still can explain most of the variability using only 7 PCs.

**Figure 8:**
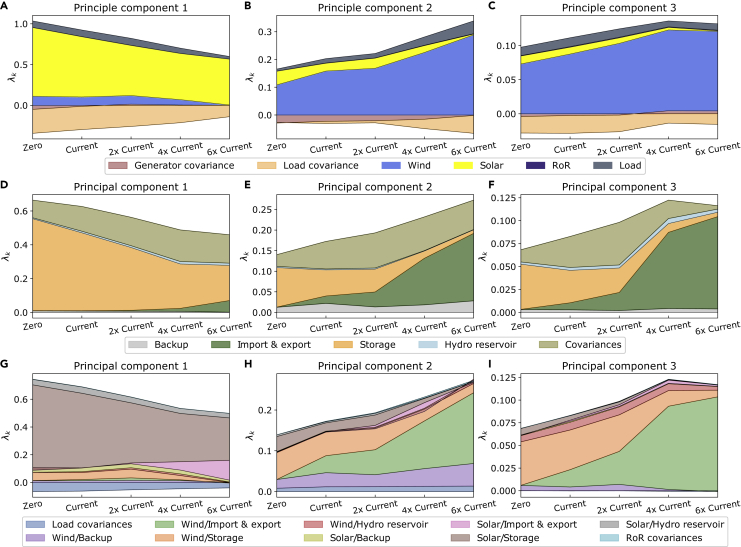
The generator contributions to the mismatch PCs, the contributions of the response terms to the mismatch PCs, and their cross-terms as a function of the transmission grid extension (A–I) In (A–C), we show the contributions of the variable renewable generators and the load together with their covariance terms for the first three PCs. Similarly, in (D–F), we show the results of the system response contributions for the first three PCs. Finally, (G–I) entail the projection on the cross-terms between the renewable generator and system response contributions for the first three PCs. Note that the covariance terms can both be positive and negative. A 95% ${\text{CO}}_{2}$ emission reduction compared to 1990 values is enforced for each of the transmission scenarios. Note that the center scenario in each panel represents the base case scenario.

We take a closer look at the transmission expansion dependence of the mismatch PCs. Figure 8 entails the variable renewable generator and load contributions to the mismatch PCs together with the responses from the system to the first three mismatch PCs. The figure maps out their dependencies on the transmission grid extension from no transmission grid capacities to six times today’s transmission volume. In (A), we see that the first PC is clearly dominated by the solar contribution. Increasing the transmission strength of the system leads to a decrease in the importance of the first PC, but it remains solar dominated. Contrary to this, (B) and (C) clearly show a dominating wind contribution. Both PCs become more important as the transmission grid is extended. In (D–F), we see the contributions of the response terms to the mismatch PCs. Naturally, the import and export contribution increases as the transmission strength is increased. For the second and third PCs, this part becomes the main contribution of the response from the network. The first PC, however, is dominated by the storage contribution. We are confirming yet again that solar power coincides with storage responses. For the cross-term contributions in (G–I), we observe which correlation effects are predominant. For the first PC, this is clearly the solar and storage interaction. There is not a strong dependence on the transmission expansion, but increasing transmission leads to a larger contribution of the interaction between solar and the injection pattern of imports and exports. For both the second and third PC, we see a clear transition from a wind/storage interaction toward wind and import/export interaction as the transmission grid is extended.

### Emission reduction effects on the mismatch

In this section, we again deviate from the base case scenario and investigate instead the effects of varying the decarbonization requirement of the system. The scenarios we present have varying amounts of ${\text{CO}}_{2}$ emission reductions compared to 1990-levels while keeping the same cross-border transmission grid strengths as the base case scenario. First, we take a look at the mismatch eigenvalue strengths as a function of the emission reduction. For this, we turn back to Figure 7. On the right side of the figure, (B) entails the results for the dependence of the eigenvalue strengths on the emission reduction relative to 1990-values. The effects are less strong than for the transmission expansion in (A), and initially only small changes in the higher order components are observed. Above 80% decarbonization, we observe a pattern where increased decarbonization strengthens the explanatory power of the first PC. However, the eigenvalue strength of the second PC decreases notably. Nevertheless, overall, the first few PCs explain roughly the same amount of the mismatch variance. For both panels, we observe that the first two PCs already explain more than 70% of the variance.

**Figure 9:**
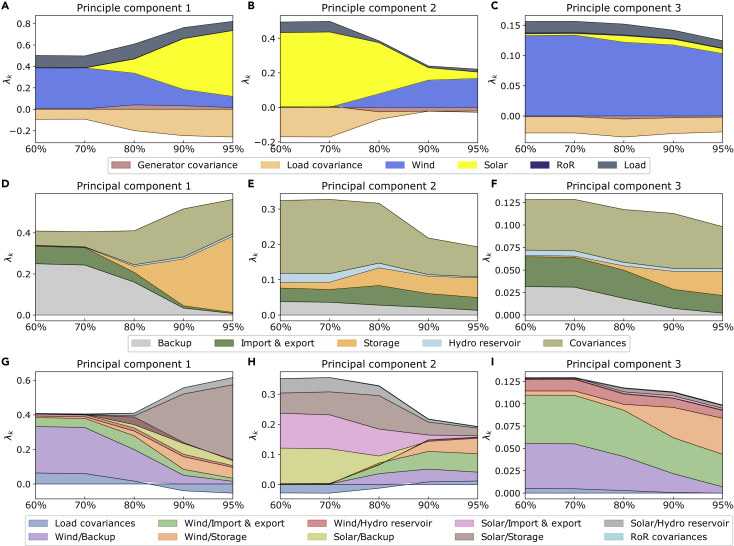
The renewable generator and load contributions to the mismatch PCs, together with the corresponding response contributions, as a function of the total emission reduction relative to 1990-values (A–C) show the generator and load contributions for the first three PCs. (D–I) In the center row of the figure, we show the response contributions to the first three PCs on (D–F), respectively. Finally, (G–I) entail the projection on the cross-terms between the renewable generator and system response contributions for the first three PCs. Note that the covariance terms can both be positive and negative. As in the base case scenario, the transmission grid is twice as strong as today’s levels in each scenario. Note that the base case scenario is represented by the rightmost scenario in each panel.

In Figure 9, we take a closer look at the emission reduction dependence of the mismatch PCs projected onto its contributions. From the top, the three rows entail the variable renewable generator and demand contributions to the mismatch PCs, the response contributions, and their interacting cross-terms, respectively. The individual panels show the results as a function of the emission reduction from a 60% decarbonization relative to 1990-levels toward the 95% reduction of our base case scenario. In (A), we observe that the first PC shifts from being wind dominated to being solar dominated when tightening the emission cap and vice versa for the second PC in (B). The third PC is clearly wind dominated. For the system response contributions visualized in the second row of the figure, we observe strong contributions by the covariances. In (D), we observe a transition from backup generation being the main contribution to the first PC toward a main contribution from the storage term as the emissions are reduced. The second PC shows only a weak dependence of the relative response contributions on the emission reduction, and the third PC is similar to the first. The second and third PCs have a relatively stable contribution from imports/exports, while for the first PCs in (D), this contribution decreases for stronger decarbonization. Let us turn to the cross-term contributions in the bottom row of panels. The decarbonization leads to a clear reduction of the relative contribution strength of the wind/backup interaction for both the first PC in (G) and the third PC in (I). For the first PC, the emission reduction leads to an increasing contribution from the solar and storage interaction. In (H), we observe a clear transition for the second PC from solar terms to the wind cross-terms, which is in line with the observations from (B). For the third PC, we observe that the decreasing wind/backup contribution is replaced by the wind/storage interaction. The contributions from the interaction of wind with imports/exports and hydro reservoirs are relatively stable.

### Effects of transmission grid extension and emission reduction on the prices

We want to investigate the effects of varying grid strengths and decarbonization levels on the price time series. The first scenarios we introduce have varying amounts of cross-border transmission grid strengths but remain at the same 95% decarbonization relative to 1990 as the base case scenario. We go from the one extreme limit with no cross-border transmission to the other extreme with a scenario that has six times the total interconnection capacity of today. Second, we look at the influence of the decarbonization level of the European electricity system in scenarios that remain at a grid extension of twice today’s levels.

Before continuing the PCA analysis, let us deviate a moment and look at the evolution of the average and variability of electricity prices shown in Figure 11. As the CO_2_ allowance is reduced, the mean price of electricity increases, but more notably the differences between the low and high quantiles increase. For the zero transmission scenario, every node needs to supply its demand at every moment which creates significant price variability. This is reduced as the transmission strengths and the network behaves more similar to a single node.

**Figure 10:**
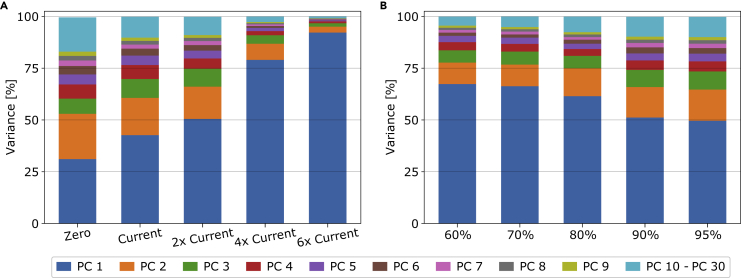
Amount of variance explained by the most dominant nodal electricity price PCs as given by their eigenvalue strengths (A and B) shows the dependence on the cross-border transmission expansion constraint. Similarly, (B) entails the dependence on the ${\text{CO}}_{2}$ emission reduction compared to 1990-levels. For each of the scenarios in (A), a 95% ${\text{CO}}_{2}$ emission reduction compared to 1990 values is enforced. In (B), the scenarios have a fixed transmission extension to twice the amount of total transmission volume of today. Note that the base case scenario is represented by the center bar in (A) and the rightmost bar in (B).

**Figure 11:**
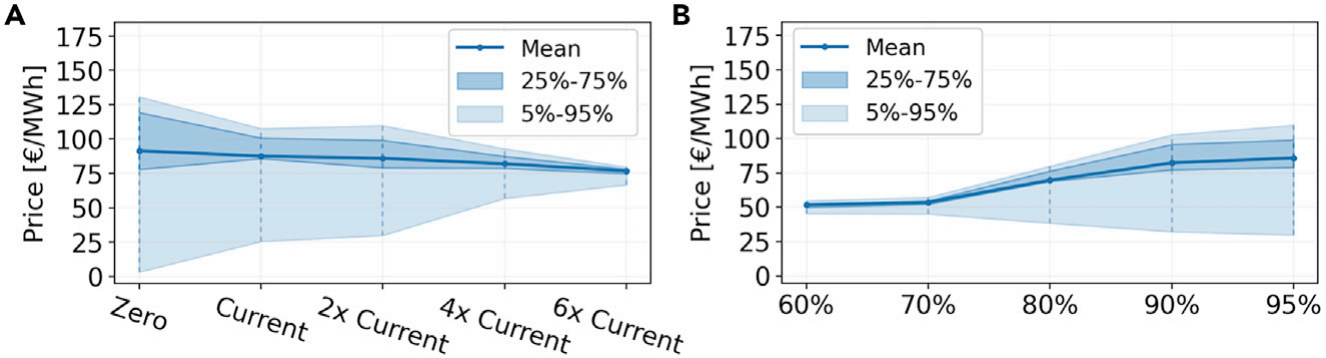
Weighted average and representative quantiles for the nodal hourly electricity price (A) shows the average prices as a function of the transmission expansion and (B) as a function of the CO_2_ emission limit. The weighted average is estimated by first calculating the average electricity price in an hour (weighting the nodal prices by each country annual demand) and subsequently calculating the average for the full year.

Returning to the PCA analysis, in (A) of Figure 10, we visualize how much of the variance in the electricity price time series is covered by the dominant PCs as a function of the cross-border transmission expansion constraint. We find an even stronger dependence on the transmission capacity than we found for the mismatch, however, now in the opposite direction. When increasing the cross-border transmission capacity, we need fewer PCs to describe the variability of the prices in Europe. This effect can be understood as the whole of Europe becoming one technical price zone as the countries are well enough connected for electricity to flow without encountering bottlenecks. (B) shows the results for the dependence of the emission reductions relative to 1990-values of the price PCA. The effects are less profound than for the transmission expansion, but we observe a clear pattern here as well. With a decreasing emission allowance, the first PC explains less of the variability. In contrast, the second, third, and fourth PCs become more important. Overall, we need slightly more PCs to explain the same amount of variance. One plausible explanation is that low-emission systems require a more diverse mix of technologies, e.g. including different storage technologies. Each of them shows a distinct operation pattern and recovers its cost differently. As a result, the price evolution in the different nodes becomes cumbersome and a higher number of PCs are needed to explain the resulting time series.

**Figure 12:**
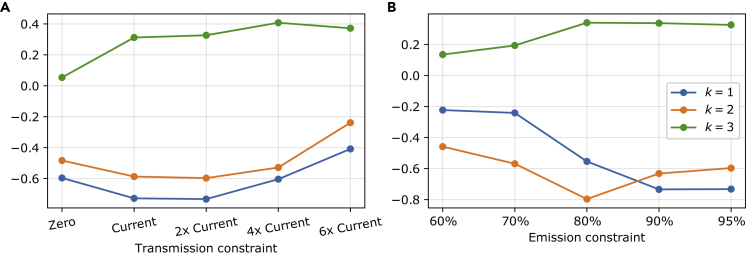
Amplitude coherence measure evolution by scenario (A and B) shows the dependence of the amplitude coherence (Equation 19) between the first three mismatch and price PCs on the transmission extension. Similarly, (B) shows the dependence on the emission reduction.

Finally, we also want to investigate how the relation between the mismatch and electricity prices depends on the transmission extension and emission reduction. We turn again to the amplitude coherence measure from Equation 19 and show the diagonal elements (i=j) for the first three PCs in Figure 12. Recall that this coherence measure quantifies the temporal overlap between the time-varying PC amplitudes of the mismatch $a_{k}^{\text{Δ}}$ and the prices $a_{k}^{\mu }$. (A) shows that the coherence is strongest around the base case scenario as both, a transmission grid reinforcement and a decrease toward no transmission lines, weakens the measure for the two dominant PCs. A similar picture is found in (B), where we see that high decarbonization leads to more coherence between the mismatch and prices for k=1, while the second component shows the most overlay at a decarbonization of 80% compared to 1990-levels.

## Conclusions

In this work, we have applied the spatiotemporally highly resolved capacity and dispatch optimization model PyPSA to investigate future renewable electricity system configurations in a networked European system. We have focused on the mismatch between the renewable generation and the demand, as the weather-driven forcing on the system, together with the dispatch decisions which can be interpreted as responses from the system on the weather-induced challenges.

Initially, by decomposing the mismatch into the generator contributions and the system responses, we confirm several effects previously described in the literature.

• Stronger transmission infrastructure leads to an increased installation of wind capacity in the cost-optimized system configuration. Hence, a larger part of the mismatch variance is explained by wind generation when transmission capacity is increased.
• Similarly, a transmission increase lowers the need for storage and increases imports and exports between the countries. This is also found to be represented in the mismatch time series.
• Solar correlates with storage capacities and their interaction significantly shapes the mismatch variability.
• Similarly, wind and transmission flows between the countries correlate and describe an increasing part of the mismatch as transmission capacities are increased.
Subsequently, a PCA approach is applied to unveil the dominant patterns in both the mismatch time series as well as the nodal electricity price time series observed in the modeled configurations.
• For the reference scenario that assumes 95% CO_2_ reduction, relative to 1990 level, and twice today’s transmission capacity, we find that the three main mismatch PCs comprise a monopole, an N-S dipole, and an E-W dipole. Together, they are enough to explain more than 85% of the mismatch variability.
• The eigenvector patterns for the first and second PCs of the nodal prices are similar to those of the mismatch PCs.
• For the nodal prices, a higher number of PCs are required to explain most of the variability since the presence of other technologies (storage, backup generators, etc) makes the nodal price patterns more complex than those of the mismatch.
• The PCA is proven to be a useful tool to explore nodal prices variability since it allows expressing complex spatiotemporal patterns with a few PCs.

### Outlook

The results in this work open several possibilities. First, PCA is commonly used as a dimensionality reduction technique and in exploratory data analysis studies like the present. It is also an excellent tool for making predictive models. Short-term forecasting energy models could be built as direct extensions of this study. Second, PCA has been used here to identify the main weather-driven mismatch patterns and a subsequent question is to what extent climate change will modify these patterns. Third, we found that the amplitude coherence (indicating the temporal correlation) between the first and the third PCs of the mismatch and nodal prices strengthen as CO_2_ emissions reduce. We learn from here that the PCA analysis becomes more relevant for future highly renewable energy systems. As this research field seems to become more relevant in the future, forthcoming complementary studies are to be expected.

### Limitations of the study

The power system is a large and complex structure. We apply a state-of-the-art optimization framework on a European power system model. Nevertheless, simplifications and approximations are necessary to keep the model within computational limits. In this regard, we should also mention that the fact that this study is based on a single weather year constitutes a limitation. Even though the specific selected weather year is found to be representative of essential weather patterns for energy systems, different weather years might influence the results slightly. Furthermore, one might want to modify the applied weather data with climate change projections. The uncertainty of the projections might though exceed their effects on the system. The European power system is modeled as an isolated system in the study at hand. In reality, the power system is intertwined with other energy sectors and including a representation of this sector coupling would give a more nuanced picture. Hence, a remaining open question is to what extent the coupling of the power system with other sectors such as heating or transport will affect the identified PCs for mismatch and nodal prices and the correlations among them.

## STAR★Methods

### Key resources table

**Table:** 

REAGENT or RESOURCE	SOURCE	IDENTIFIER
**Software and algorithms**		

Figure source code: mismatch and price PCA in a highly-decarbonised networked European power system	https://zenodo.org/record/6472962	https://doi.org/10.5281/zenodo.6472962

### Resource availability

#### Lead contact

Further information and requests for resources should be directed to and will be fulfilled by the Lead Author, Leon J. Schwenk-Nebbe (leonsn@mpe.au.dk).

#### Materials availability

This study did not generate new materials.

#### Data and code availability

• Data: All data reported in this paper will be shared by the lead contact upon request.
• Code: All original code reported in this paper for the analysis and figure creating is openly available at https://zenodo.org/record/6472962 with https://doi.org/10.5281/zenodo.6472962.
• Any additional information required to reanalyse the data reported in this paper is available from the lead contact upon request.

### Method details

In the following, we present further detail on the applied model, its main output of interest for the study at hand, and shortly give an overview of the applied statistical tools.

#### Modeling the European power system

In the present study, we apply a modern and open energy system optimisation framework, PyPSA (Brown et al., 2018a), to comprehensively, thoroughly and adequate-detailed model the European electricity system of the future. The applied model was first introduced in (Brown et al., 2018b) and successively extended. The specific version of the model applied here was introduced in (Victoria et al., 2019). The model consists of 30 nodes, each one representing a European country. It includes the 27 European Union member states except for Malta and Cyprus and includes Norway, the United Kingdom, Switzerland, Serbia, and Bosnia and Herzegovina. The transmission lines between countries are approximated as single cross-border lines to stay within computational limits and because the study at hand is limited to country-level analyses. The objective of the model is to minimise the total annualised system cost of the whole European electricity system. The problem is formulated as a linear function subject to technical and physical constraints. A simplified formulation of the optimisation problem is shown in Equation (1) where *n* represents every country and *t* every hour in a year.

(Equation 1) $$\begin{array}{l} \text{min}\sum_{n,t}\left(\text{capital}\_{\text{cost}}_{n}+\text{operational}\_{\text{cost}}_{n}\left(t\right)\right)\\ \text{s}.\text{t}.\\ {\text{generation}}_{n}\left(t\right)+{\text{balancing}}_{n}\left(t\right)={\text{demand}}_{n}\left(t\right)\;↔\;\mu_{n}\left(t\right)\\ \sum \text{emissions}\leq {\text{CAP}}_{{\text{CO}}_{2}}\;↔\;\mu_{{\text{CO}}_{2}} \end{array}$$

The objective function hence includes the annualised capital costs of all system components, which encompasses generators, converters, storage power capacities, storage energy capacities, transmission line capacities, and variable costs of generators and storage dispatch, and energy conversions. Electricity can be produced by solar PV, onshore and offshore wind, hydro and Open Cycle Gas Turbine (OCGT) units. It can be stored in batteries and H_2_ storage. The cost, efficiency and lifetime assumed for different technologies can be seen in Tables 1 and 2 in (Victoria et al., 2019).

We assume no variable cost for the transmission grid. The model performs a linearised optimal power flow calculation. The objective function (1) is subject to various constraints. Most elementary, we have the nodal energy balance constraint that requires the demand at each country node *n* to be met at each hourly time step *t* of the year by local generation, storage, or transmission. The Lagrange multiplier $\mu_{n}\left(t\right)$ associated with the nodal energy balance constraint represents the nodal marginal electricity price. It is noteworthy that large prices in the model time series are an indication of challenging period for the system. Other constraints in the model include that the maximum power flow through the transmission lines is limited by their physical dimensions, that the renewable generation capacity can only be placed on suitable lands, and the emission constraint that limits the maximum ${\text{CO}}_{2}$ emissions. Associated to the latter, we have the Lagrange multiplier $\mu_{{\text{CO}}_{2}}$ which represents the necessary price of ${\text{CO}}_{2}$ emissions that would be required to avoid surpassing the ${\text{CO}}_{2}$ emission cap in an open market. For the mathematical formulation of the optimisation problem, see Appendix A in (Victoria et al., 2019) which describes the model in full detail.

#### Mismatch definition and cross-correlations

We conveniently rewrite the dispatch equations used in the PyPSA model as equations describing the mismatch between the variable renewable generation and the inelastic demand:

(Equation 2) $$\vec{\text{Δ}}\left(t\right)={\vec{G}}^{W}\left(t\right)+{\vec{G}}^{S}\left(t\right)+{\vec{G}}^{RoR}\left(t\right)-\vec{L}\left(t\right).$$

The mismatch vector $\vec{\text{Δ}}\left(t\right)=\sum_{n}{\text{Δ}}_{n}\left(t\right){\vec{e}}_{n}$ is defined as the difference between variable renewable generation from wind ($G^{W}$), solar ($G^{S}$), run-of-river ($G^{RoR}$) generation, and the load (*L*). It is also known as the negative net load. We write the quantities as vectors as they have a component ${\text{Δ}}_{n}$ for each country *n*, and we denote the unit vector ${\vec{e}}_{n}$. When referring to the renewable generation in this study, we refer to the dispatched renewable generation and not the bare generation that would be achievable from the installed capacity and weather. Another way of thinking about this is that the curtailment is already subtracted from the renewable generation. We argue that the renewable generation that needs to be curtailed in real life never reaches the grid as capacities are throttled beforehand. We show the amount of curtailment on Figure S1.

We express the variance of the mismatch as follows:

(Equation 3) $$\text{Var}\left(\vec{\text{Δ}}\left(t\right)\right)=\sum_{n}\text{Var}\left({\text{Δ}}_{n}\left(t\right)\right)=\text{Var}\left({\vec{G}}^{W}\left(t\right)\right)+\text{Var}\left({\vec{G}}^{S}\left(t\right)\right)+\text{Var}\left({\vec{G}}^{RoR}\left(t\right)\right)+\text{Var}\left(\vec{L}\left(t\right)\right)+2\text{Cov}\left({\vec{G}}^{W}\left(t\right),{\vec{G}}^{S}\left(t\right)\right)+2\text{Cov}\left({\vec{G}}^{W}\left(t\right),{\vec{G}}^{RoR}\left(t\right)\right)+2\text{Cov}\left({\vec{G}}^{S}\left(t\right),{\vec{G}}^{RoR}\left(t\right)\right)-2\text{Cov}\left({\vec{G}}^{W}\left(t\right),\vec{L}\left(t\right)\right)-2\text{Cov}\left({\vec{G}}^{S}\left(t\right),\vec{L}\left(t\right)\right)-2\text{Cov}\left({\vec{G}}^{RoR}\left(t\right),\vec{L}\left(t\right)\right).$$

The weather-induced mismatch evokes a combination of responses from the system. The mismatch can also be expressed as the feedback of the system. This response consists of the nodal storage charge/discharge operation (*S*), the import/export injection pattern (*P*), the hydro reservoir dispatch (*H*), and the gas-fuelled backup generation (*B*):

(Equation 4) $$\vec{\text{Δ}}\left(t\right)=\vec{S}\left(t\right)+\vec{P}\left(t\right)+\vec{H}\left(t\right)+\vec{B}\left(t\right).$$

The storage term includes batteries, hydrogen storage (with fuel cells and electrolysers), and pumped hydro storage. Backup generation in the model is assumed to be open-cycle gas turbines. Henceforward, we also express the mismatch variance in terms of responses from the system:

(Equation 5) $$\text{Var}\left(\vec{\text{Δ}}\left(t\right)\right)=\text{Var}\left(\vec{S}\left(t\right)\right)+\text{Var}\left(\vec{P}\left(t\right)\right)+\text{Var}\left(\vec{H}\left(t\right)\right)+\text{Var}\left(\vec{B}\left(t\right)\right)+2\text{Cov}\left(\vec{S}\left(t\right),\vec{P}\left(t\right)\right)+2\text{Cov}\left(\vec{S}\left(t\right),\vec{H}\left(t\right)\right)+2\text{Cov}\left(\vec{S}\left(t\right),\vec{B}\left(t\right)\right)+2\text{Cov}\left(\vec{P}\left(t\right),\vec{H}\left(t\right)\right)+2\text{Cov}\left(\vec{P}\left(t\right),\vec{B}\left(t\right)\right)+2\text{Cov}\left(\vec{H}\left(t\right),\vec{B}\left(t\right)\right).$$

The variance of the mismatch can also be written as a combination of the cross terms between the two expressions in Equations (2) and (4):

(Equation 6) $$\begin{array}{l} \text{Var}\left(\vec{\text{Δ}}\left(t\right)\right)=\sum \text{Cov}\left(\vec{X}\left(t\right),\vec{Y}\left(t\right)\right)\\ \text{for}\;X\in \left\{G^{W},G^{S},G^{RoR},-L\right\}\\ \text{and}\;Y\in \left\{S,P,H,B\right\}. \end{array}$$

We will apply all three different expressions to investigate which system components affect the mismatch characteristics.

#### Principal component analysis

A Principal Component Analysis (PCA) decomposes the variance of the nodal mismatches and the nodal marginal electricity prices into coherent spatio-temporal patterns. Most of the variance can then be explained by only a few strongest of such patterns. PCA is based on the covariance matrix $Cov\left({\text{Δ}}_{m},{\text{Δ}}_{n}\right)$. The associated eigenvectors ${\vec{p}}_{k}$ describes the coherent patterns, and the eigenvalues $\lambda_{k}$ represents their strength.

The mismatch $\vec{\text{Δ}}\left(t\right)$ can be expressed as linear combination of the standard basis vectors ${\vec{e}}_{n}$ or the PCA eigenvectors:

(Equation 7) $$\vec{\text{Δ}}\left(t\right)=\sum_{n}{\text{Δ}}_{n}\left(t\right)\;{\vec{e}}_{n}={\left\langle \vec{\text{Δ}}\left(t\right)\right\rangle }_{t}+\sqrt{\text{Var}\left(\vec{\text{Δ}}\left(t\right)\right)}\sum_{k}a_{k}^{\text{Δ}}\left(t\right)\;{\vec{p}}_{k}^{\text{Δ}}.$$

The eigenvalues are given as the time-averaged square of the principal component (PC) amplitudes, following (Haken, 1996):

(Equation 8) $$\lambda_{k}=\left\langle a_{k}{\left(t\right)}^{2}\right\rangle \;.$$

The eigenvalues sum up to $\underset{k}{\sum }\lambda_{k}=1$.

Similarly, we can also perform the PCA on the price time series:

(Equation 9) $$\vec{\mu }\left(t\right)={\left\langle \vec{\mu }\left(t\right)\right\rangle }_{t}+\sqrt{\text{Var}\left(\vec{\mu }\left(t\right)\right)}\sum_{k}a_{k}^{\mu }\left(t\right)\;{\vec{p}}_{k}^{\mu }.$$

$\vec{\mu }\left(t\right)$ represent the prices in energy-only wholesale markets. The long-term market equilibrium imposed in the model requires that for some hours, the nodal electricity prices are higher than the variable cost of the most expensive generators, ensuring that they can also recover their capital cost. The optimiser finds particular solutions comprising a few time steps with extremely high prices. In some real markets, these price spikes are avoided by setting a cap in the electricity prices and compensating backup generators via capacity mechanisms. In our results, we filter the price peak to avoid that they mask more meaningful information. Hence, we perform a simple filtering by applying a cut-off value by letting $\mu_{n}\left(t\right)\leq 1000$ /€ MWh before performing the PCA. This filtering only affects very few hours of the year.

#### Projections

On top of the Principal Component Analysis, we also want to find out how much each principal mismatch/price component contributes to the various (renewable) generation and dispatch technologies.

We begin by defining the projections for the generator and load contributions. In Equation (2) we have defined the mismatch $\vec{\text{Δ}}\left(t\right)$ as the difference between the variable renewable generation from wind $G^{W}$, solar $G^{S}$, run of river $G^{RoR}$ generation, and the load *L*. We insert this expression into Equation (7) and perform the dot product with the orthogonal eigenvectors ${\vec{p}}_{k}^{\text{Δ}}$. At the same time, we can use our definition from Equation (4) and define the mismatch in terms of the backup generation *B*, injection pattern *P*, hydro reservoir yield *H*, and storage operation *S*, and perform the same calculation. These two approaches then yield the following two expressions:

(Equation 10) $$a_{k}^{\text{Δ}}\left(t\right)=\frac{1}{\sqrt{\text{Var}\vec{\text{Δ}}\left(t\right)}}\left(\vec{\text{Δ}}\left(t\right)-\left\langle \vec{\text{Δ}}\right\rangle \right)\cdot {\vec{p}}_{k}=\frac{1}{\sqrt{\text{Var}\vec{\text{Δ}}\left(t\right)}}\left(G_{k}^{W}\left(t\right)+G_{k}^{S}\left(t\right)+G_{k}^{RoR}\left(t\right)-L_{k}\left(t\right)\right)=\frac{1}{\sqrt{\text{Var}\vec{\text{Δ}}\left(t\right)}}\left(B_{k}\left(t\right)+P_{k}\left(t\right)+H_{k}\left(t\right)+S_{k}\left(t\right)\right),$$

where

(Equation 11) $$\begin{array}{l} G_{k}^{W}\left(t\right)=\left({\vec{G}}^{W}\left(t\right)-\left\langle G^{W}\right\rangle \right)\cdot {\vec{p}}_{k},\\ \vdots \\ S_{k}\left(t\right)=\left(\vec{S}\left(t\right)-\left\langle S\right\rangle \right)\cdot {\vec{p}}_{k}. \end{array}$$

By applying Equation (8) to the first expression in Equation (10) we arrive at a definition for the eigenvalues (omitting the Δ-superscript for clarity):

(Equation 12) $$\lambda_{k}=\frac{1}{\text{Var}\left(\vec{\text{Δ}}\left(t\right)\right)}\left(\left\langle G_{k}^{W}{\left(t\right)}^{2}\right\rangle +\left\langle G_{k}^{S}{\left(t\right)}^{2}\right\rangle +\left\langle G_{k}^{RoR}{\left(t\right)}^{2}\right\rangle +\left\langle L_{k}{\left(t\right)}^{2}\right\rangle +2\left\langle G_{k}^{W}\left(t\right)G_{k}^{S}\left(t\right)\right\rangle +2\left\langle G_{k}^{W}\left(t\right)G_{k}^{RoR}\left(t\right)\right\rangle +2\left\langle G_{k}^{S}\left(t\right)G_{k}^{RoR}\left(t\right)\right\rangle -2\left\langle L_{k}\left(t\right)G_{k}^{W}\left(t\right)\right\rangle -2\left\langle L_{k}\left(t\right)G_{k}^{S}\left(t\right)\right\rangle -2\left\langle L_{k}\left(t\right)G_{k}^{RoR}\left(t\right)\right\rangle \right)$$

We can also choose to write this decomposition as

(Equation 13) $$\lambda_{k}=\lambda_{k}^{W}+\lambda_{k}^{S}+\lambda_{k}^{RoR}+\lambda_{k}^{L}+\lambda_{k}^{WS}+\lambda_{k}^{WRoR}+\lambda_{k}^{SRoR}-\lambda_{k}^{L\;W}-\lambda_{k}^{L\;S}-\lambda_{k}^{L\;RoR},$$

where

(Equation 14) $$\begin{array}{l} \lambda_{k}^{XY}=\frac{2-\delta_{XY}}{\text{Var}\left(\vec{\text{Δ}}\left(t\right)\right)}\left(X_{k}\left(t\right)-\left\langle X_{k}\right\rangle \right)\left(Y_{k}\left(t\right)-\left\langle Y_{k}\right\rangle \right)\\ \text{for}\;X,Y\in \left\{G^{W},G^{S},G^{RoR},-L\right\}. \end{array}$$

With these projections, we find the contribution of the generator terms and the load to the eigenvalue.

Next, we define the projections for the system response contributions in a similar fashion. For this, we apply that we defined the mismatch in terms of the responses from storage *S*, import/export *P*, hydro reservoir *H*, and fossil backup generation *B* in the second part of Equation (10). Following the same derivation as before, and writing the eigenvalue in terms of individual response terms as

(Equation 15) $$\lambda_{k}=\lambda_{k}^{B}+\lambda_{k}^{P}+\lambda_{k}^{H}+\lambda_{k}^{S}+\lambda_{k}^{BP}+\lambda_{k}^{BH}+\lambda_{k}^{BS}+\lambda_{k}^{PH}+\lambda_{k}^{PS}+\lambda_{k}^{HS},$$

we arrive at our response eigenvalue projections:

(Equation 16) $$\begin{array}{l} \lambda_{k}^{XY}=\frac{2-\delta_{XY}}{\text{Var}\left(\vec{\text{Δ}}\left(t\right)\right)}\left(X_{k}\left(t\right)-\left\langle X_{k}\right\rangle \right)\left(Y_{k}\left(t\right)-\left\langle Y_{k}\right\rangle \right)\\ \text{for}\;X,Y\in \left\{B,P,H,S\right\}. \end{array}$$

With these projections, we find the contribution of the dispatch terms to the eigenvalue.

The eigenvalues (8) can also be expressed as the cross terms between the two expressions in Equation (10). Using this, and again following an analogous derivation with the eigenvalues expressed as the cross-terms we arrive at our third projection:

(Equation 17) $$\begin{array}{c} \lambda_{k}=\sum_{X,Y}\lambda_{k}^{XY},\\ \lambda_{k}^{XY}=\frac{1}{\text{Var}\left(\vec{\text{Δ}}\left(t\right)\right)}\left(X_{k}\left(t\right)-\left\langle X_{k}\right\rangle \right)\left(Y_{k}\left(t\right)-\left\langle Y_{k}\right\rangle \right), \end{array}$$

where $X\in \left\{G^{W},G^{S},G^{RoR},-L\right\}$ and Y∈{B,P,H,S}. We apply the Equations (14), (16), and (17) in to portray the individual contributions to both the mismatch and price PCs.

#### Coherence measures

We are also interested in the connection between the PCs of the mismatch and the PCs of the nodal electricity prices. To this end, we introduce two coherence measures. In The coherence of mismatch and price PCs, we apply these measures to investigate the coherence between the investigated PCs from the base scenario. The first measure is a simple measure of orthogonality that we denote the eigenvector coherence:

(Equation 18) $$c_{i,j}^{\left(1\right)}=\left|{\vec{p}}_{k_{i}}^{\text{Δ}}\cdot {\vec{p}}_{k_{j}}^{\mu }\right|.$$

A second coherence measure analyses the alignment of the time-varying PC amplitudes. We denote this measure the amplitude coherence and define it as follows:

(Equation 19) $$c_{i,j}^{\left(2\right)}=\frac{1}{\sqrt{\left\langle a_{k_{i}}^{\text{Δ}2}\right\rangle \left\langle a_{k_{j}}^{\mu 2}\right\rangle }}\left\langle a_{k_{i}}^{\text{Δ}}a_{k_{j}}^{\mu }\right\rangle .$$

The amplitude coherence thereby quantifies the temporal overlap between the time-varying PC amplitudes $a_{k}\left(t\right)$ of the mismatch and the marginal prices.
