# Supplementary material for: Principal spatiotemporal mismatch and electricity price patterns in a highly decarbonized networked European power system
Source: iScience. 2022 May 10;25(6):104380. doi: 10.1016/j.isci.2022.104380 (PMC9126784; doi:10.1016/j.isci.2022.104380)
Supplement: Document S1. Figures S1–S8 and Tables S1–S5 [file mmc1.pdf]

iScience, Volume *25*

Supplemental information

Principal spatiotemporal mismatch and electricity price patterns in a highly decarbonized networked European power system

Leon Joachim Schwenk-Nebbe, Jonas Emil Vind, August Jensen Backhaus, Marta Victoria, and Martin Greiner

Supplemental Materials

Curtailment of Renewable Generation

The curtailment of renewable generation has been subtracted from the individual generator components in Equation (2). In a highly renewable electricity system, the curtailment of renewable generation becomes necessary as the potential generation exceeds the demand, transmission, and storage capabilities of the system. We visualise the combined curtailment from wind, solar, and run-of-river generation in Figure S1. The figure includes multiple scenarios. The light blue curve

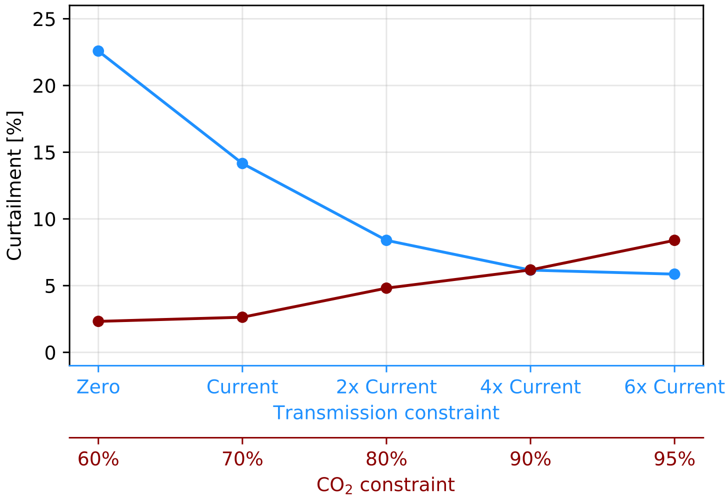

Figure S1: **Total curtailment of renewable generation relative to the load is shown for various scenarios, related to STAR Methods**. For the transmission extension scenarios (top axis), a CO_2 reduction of 95% compared to 1990 levels is assumed, while a two-times current transmission capacities expansion is assumed for the scenarios with varying emissions (bottom axis). Hence, the centre case on the (light blue) transmission expansion curve coincides with the rightmost scenario on the (dark red) emission reduction curve.

shows the curtailment as a function of the transmission extension for the highly decarbonised scenarios that require emissions to be reduced by 95% compared to 1990-values. Without any cross-border transmission grid, the countries need to install large amounts of renewable capacity to supply their demand. As transmission is increased, the variable renewable generation can be exported more freely, and the renewable capacity is utilised more, leading to less curtailment. The dark red curve shows the curtailment as a function of the emissions reduction parameter for systems with a transmission capacity of twice today's system. Not surprisingly, the curtailment increases as emissions are reduced. This is because the share of renewable generation increases as further decarbonisation requires a larger instalment of renewable capacity. This effect cannot be fully counterbalanced by increasing storage capacities.

Extended Principal Component Investigation

Supplemental to the main article we include additional tables and figures in this section. The first three to four PCs already entail most of the variability in the time series of both the analysed

mismatch and electricity prices. For the sense of completeness, we present the results for the remaining PCs up to the sixth PC in this section.

In relation to Section 2.2, see Table S1 for an overview of the seven most dominant mismatch PCs with which we explain more than 95% of the variance of the mismatch time series. Similarly, for Section 2.3, see Table S2 which contains the fifteen most dominant price PC eigenvalue strengths needed to explain 95% of the variance of the country-specific electricity price time series.

PC	Variance [%]	Cumulative variance [%]
1	56.3	56.3
2	19.3	75.6
3	9.8	85.4
4	4.9	90.3
5	2.7	93.0
6	1.8	94.8
7	1.3	96.1

Table S1: **The amount of the variance explained by the mismatch PCs shown alongside the cumulative variance, related to Figure 3**. With 7 PCs we are able to explain more than 95% of the variance in our original data. See also Figure 3 for a graphical representation of the first six mismatch PC eigenvectors and Table S2 for a similar table on the price PCs.

PC	Variance [%]	Cumulative variance [%]
1	49.6	49.6
2	15.1	64.7
3	8.8	73.4
4	4.9	78.3
5	3.7	82.0
6	2.7	84.7
7	2.1	86.8
8	1.7	88.5
9	1.5	90.0
10	1.3	91.3
11	1.1	92.4
12	0.9	93.3
13	0.8	94.1
14	0.7	94.9
15	0.6	95.5

Table S2: **The amount of the variance explained by the nodal electricity price PCs shown alongside the cumulative explained variance, related to Figure 5**. With 15 PCs we are able to explain 95% of the variance. For a graphical representation of the first six PCs of the prices see Figure 5.

In the main text we presented the spatial structure of the first four PCs for the base case scenario and supplement this here by the fifth and sixth PC for both the mismatch and prices. For the mismatch PCA, this is shown in Figure S2. We observe more complex patterns emerging after the first three PCs of the mismatch. These have the main emphasis on peripheral countries in the

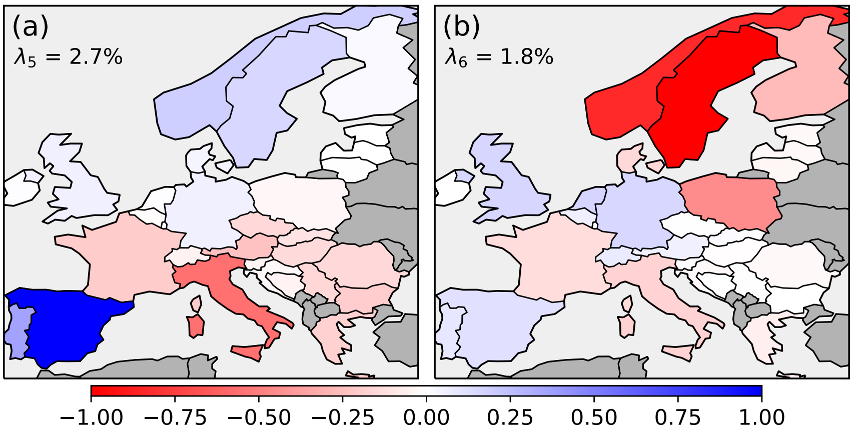

Figure S2: **Main PCA projections of the mismatch between renewable generation and demand, related to Figure 3**. On each of the Panels (a) and (b) we show a map visualising the mismatch PCs 5 and 6, respectively, for the base case scenario. The panels also indicate the associated eigenvalue strengths λ_k. See also Figure 3 for the first four PCs.

network. For PC 4 we observed that the United Kingdom was the strongest driver and similarly for PC 5 we find Spain in this role whereas for PC 6 Norway and Sweden are the strongest drivers. The patterns of PC 4 and 5 could be described loosely as north-central-south tripoles. Similarly, we show the fifths and sixth PC of the electricity prices in the base case scenario in Figure S3.

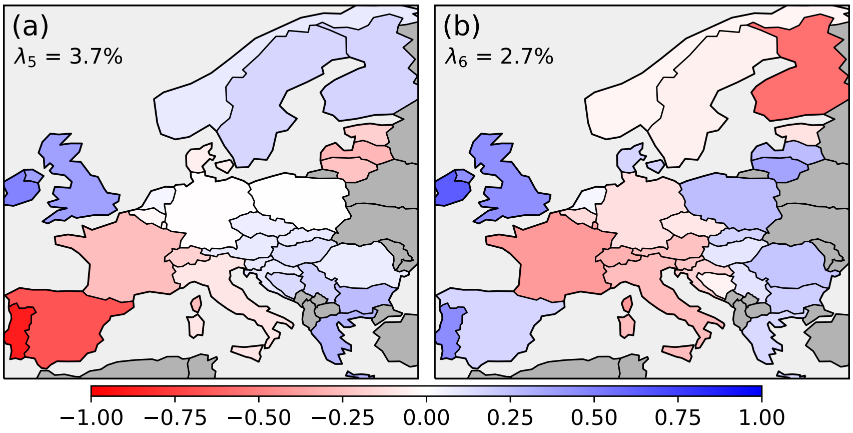

Figure S3: **Main PCA projections of the electricity price time series, related to Figure 5**. On each of the Panels (a) and (b) we show a map visualising the price PCs 5 and 6, respectively, for the base case scenario. The panels also indicate the associated eigenvalue strengths λ_k. See also Figure 5 for the first four PCs.

Supplemental to Section 2.4, we show the results from the eigenvector coherence measure from Equation (18) also for the fifth and sixth PC in Table S3. Similar to the strong correlation between the first PC of the mismatch and the first PC of the prices and the second mismatch PC with the second price PC, we also observe a strong correlation for the fifth mismatch PC with the fifth price PC. To understand this we can compare the mismatch PC patterns of Figure 3 with the price PCs in Figure 5 by looking at Panel (e) on both figures. On the two figures, we

Price	Mismatch PC 1	PC 2	PC 3	PC 4	PC 5	PC 6
PC 1	0.588	0.101	0.027	0.074	0.129	0.152
PC 2	0.172	0.467	0.146	0.177	0.134	0.152
PC 3	0.311	0.180	0.275	0.111	0.112	0.371
PC 4	0.214	0.258	0.264	0.239	0.198	0.122
PC 5	0.240	0.208	0.108	0.351	0.518	0.148
PC 6	0.142	0.020	0.092	0.335	0.241	0.108

Table S3: **Eigenvector coherence measure, related to Table 1**. Showing the eigenvector coherence between the principal components (PCs) of the mismatch and the nodal electricity prices for the baseline scenario as computed by the coherence measure from Equation (18). Note that the cell background colour intensity corresponds to the cell values for convenient comparison.

see that Spain is constituting the most significant contribution in the two cases but there is no further obvious similarity.

Furthermore, we also show the results from introducing a weighting to our eigenvector coherence. The eigenvector coherence from Equation (18), introduced in Section 5.3.5, is unweighted as the importance of the individual PCs is taken to be the same. In practice, the importance of the individual PCs depends on their eigenvalue strength. Therefore, we include the time-averaged eigenvalues λ_k into our measure. This forms another coherence measure:

$$c_{i,j}^{(3)} = \sqrt{\lambda_{k_i}^{\Delta} \lambda_{k_j}^{\mu}} \left| \vec{p}_{k_i}^{\Delta} \cdot \vec{p}_{k_j}^{\mu} \right| . \tag{S1}$$

The results from this measure are shown in Table S4, again for the first six PCs. The table

Price	Mismatch PC 1	PC 2	PC 3	PC 4	PC 5	PC 6
PC 1	0.311	0.031	0.006	0.011	0.015	0.014
PC 2	0.050	0.080	0.018	0.015	0.009	0.008
PC 3	0.069	0.023	0.026	0.007	0.005	0.015
PC 4	0.035	0.025	0.018	0.012	0.007	0.004
PC 5	0.035	0.018	0.007	0.015	0.016	0.004
PC 6	0.017	0.001	0.005	0.012	0.006	0.002

Table S4: **Weighted coherence measure, related to Table 1**. Showing the correlation between the mismatch and nodal electricity prices for the baseline scenario as computed by the weighted coherence measure from Equation (S1). Note that the cell background colour intensity corresponds to the cell values.

shows that the weighting coefficient results in a strong decline in the value of the measure. The correlation between the two first PCs of the mismatch and prices is remaining significant but the other correlations turn out to be weak. This is due to the decreasing explanatory power of the PCs as their eigenvalues become small. Besides a significant correlation between the PC1 and PC1, PC2 and PC2 of the mismatch and price, respectively, we also see a correlation between the first mismatch PC and the third price PC. This correlation is stronger than the correlation between the third mismatch PC with the third price PC. Comparing the structural composition of

the mismatch PCs in Figure 3 with the price PCs in Figure 5 this result makes intuitively sense. The same behaviour can also be observed in Table 1 but becomes more eye-catching here with the weighting parameter applied.

We also show the results of the amplitude coherence from Equation (19) for the first six PCs in Table S5. Recall that this coherence measure compares the temporal structure of the mismatch

	Mismatch					
Price	PC 1	PC 2	PC 3	PC 4	PC 5	PC 6
PC 1	-0.733	0.317	0.076	0.022	-0.100	-0.131
PC 2	-0.272	-0.597	0.162	-0.196	-0.120	0.251
PC 3	0.240	-0.226	0.326	-0.095	-0.112	-0.445
PC 4	-0.251	-0.293	-0.341	0.341	0.073	-0.138
PC 5	-0.163	-0.121	-0.114	-0.413	0.383	-0.043
PC 6	0.012	0.035	-0.118	-0.397	-0.186	0.017

Table S5: **Amplitude coherence measure, related to Table 2**. Showing the amplitude coherence between different PC amplitudes as calculated by the measure defined in Equation (19). We are comparing the PC amplitudes of the mismatch and the nodal electricity prices for the baseline scenario. Note that the cell background colour intensity corresponds to the cell values with blue tones for positive values and red for negative values. (For interpretation of the references to colour in this table legend, the reader is referred to the web version of this article.)

and price PC amplitudes.

Complementary to the results shown in Section 2.2 we show similar visualisations for the third to sixth most-relevant PCs in Figure S4 and the corresponding generator and response contributions in Figure S6.

The results for the PCs three through six are visualised in Figure S6. Like for PC two, we find here that the PCs three, four, and six are clearly wind dominated while the PC five is solar dominated but also has a significant wind contribution. In terms of responses, the picture is more mixed for the third and fourth PC. The response contribution of the fifth PC is again driven by balancing interactions with the storages. Lastly, the response contribution of the sixth PC is by hydro reservoir, imports and exports, and their interacting cross term.

For the prices analysed in Section 2.3 we similarly show the third to sixth PCs in Figure S5.

Supplemental to Section 3.1 we show also the results for the PCs four through six. The top row in Figure S7 entails the variable renewable generator and load contributions to the PCs, while the bottom row shows how the individual responses from the system contribute to the fourth, fifth, and sixth mismatch PC. Analogous to Figure 8, the figure maps out their dependencies on the transmission grid extension. On Panel (a), and (c) we see that the fourth and sixth PCs are clearly dominated by wind contributions while the fifth PC on Panel (b) is dominated by solar contribution. Increasing the transmission strength of the system has little effect on the relative importance of the generator contributions. On the Panels (d) through (f), we see the contributions of the response terms to the mismatch PCs. Naturally, the import and export contribution increases as the transmission strength is increased. For the three shown PCs this part becomes the main contribution of the response from the network as transmission is increased. The second PC has again a large storage contribution. Like the first PC, this large storage response

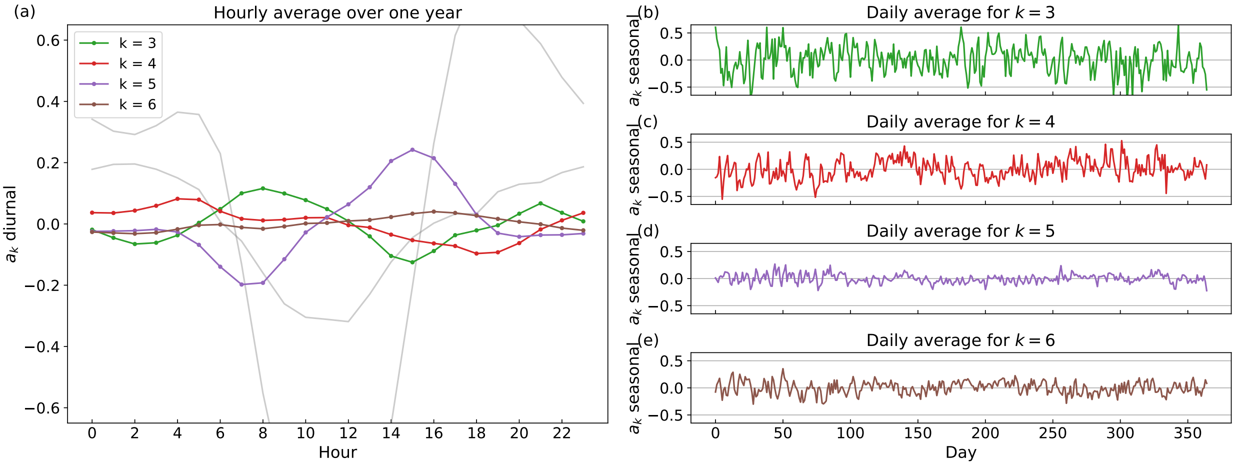

Figure S4: **Mismatch PCA overview for PCs 3 to 6, related to Figure 4**. Analogous to Figure 4, we show here the same visualisations for the mismatch PCs 3, 4, 5, and 6. Panel (a) shows the diurnal behaviour of the mismatch PCs by showing the hourly averaged values of the PC amplitudes throughout one year. Panels (b-e) visualise the corresponding seasonal behaviours of the mismatch PCs by showing the daily averages of the PC amplitudes 3 to 6, respectively. Note that the individual PCs have the same colour in the different panels.

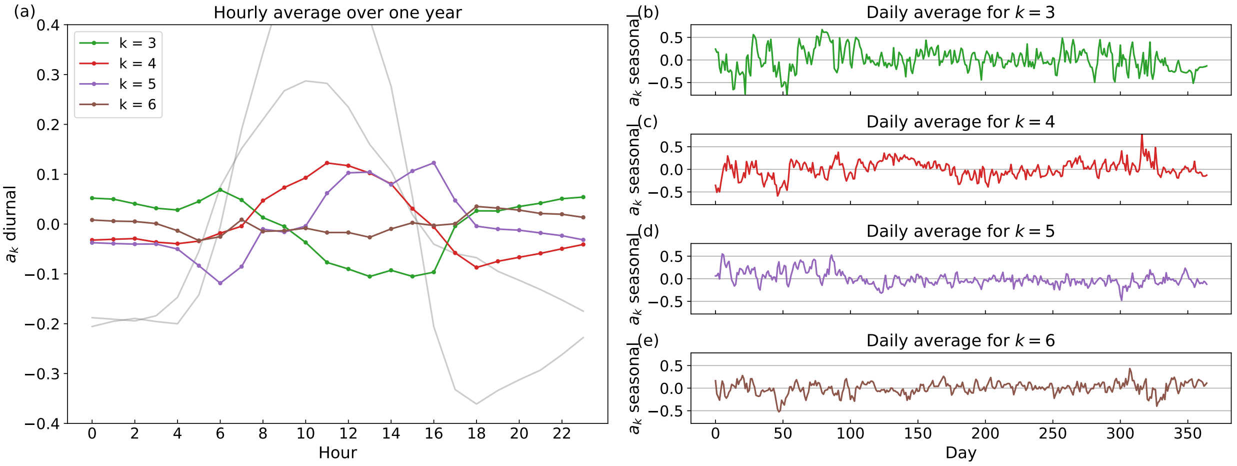

Figure S5: **Price PCA overview for PCs 3 to 6, related to Figure 6**. Analogous to Figure 6, we show here the same visualisations for the nodal electricity price PCs 3, 4, 5, and 6. Panel (a) shows the diurnal behaviour of the price PCs by showing the hourly averaged values of the PC amplitudes throughout one year. Panels (b-e) visualise the corresponding seasonal behaviours of the price PCs by showing the daily averages of the PC amplitudes 3 to 6, respectively. Note that the individual PCs have the same colour in the different panels.

coincides again with a large solar contribution. The third PC shows also large hydro reservoir contributions and a large but negative contribution from the covariance terms. Figure S8 entails the variable renewable generator and load contributions to the fourth to sixth mismatch PCs, as well as the corresponding response contributions, as a function of the emission reduction.

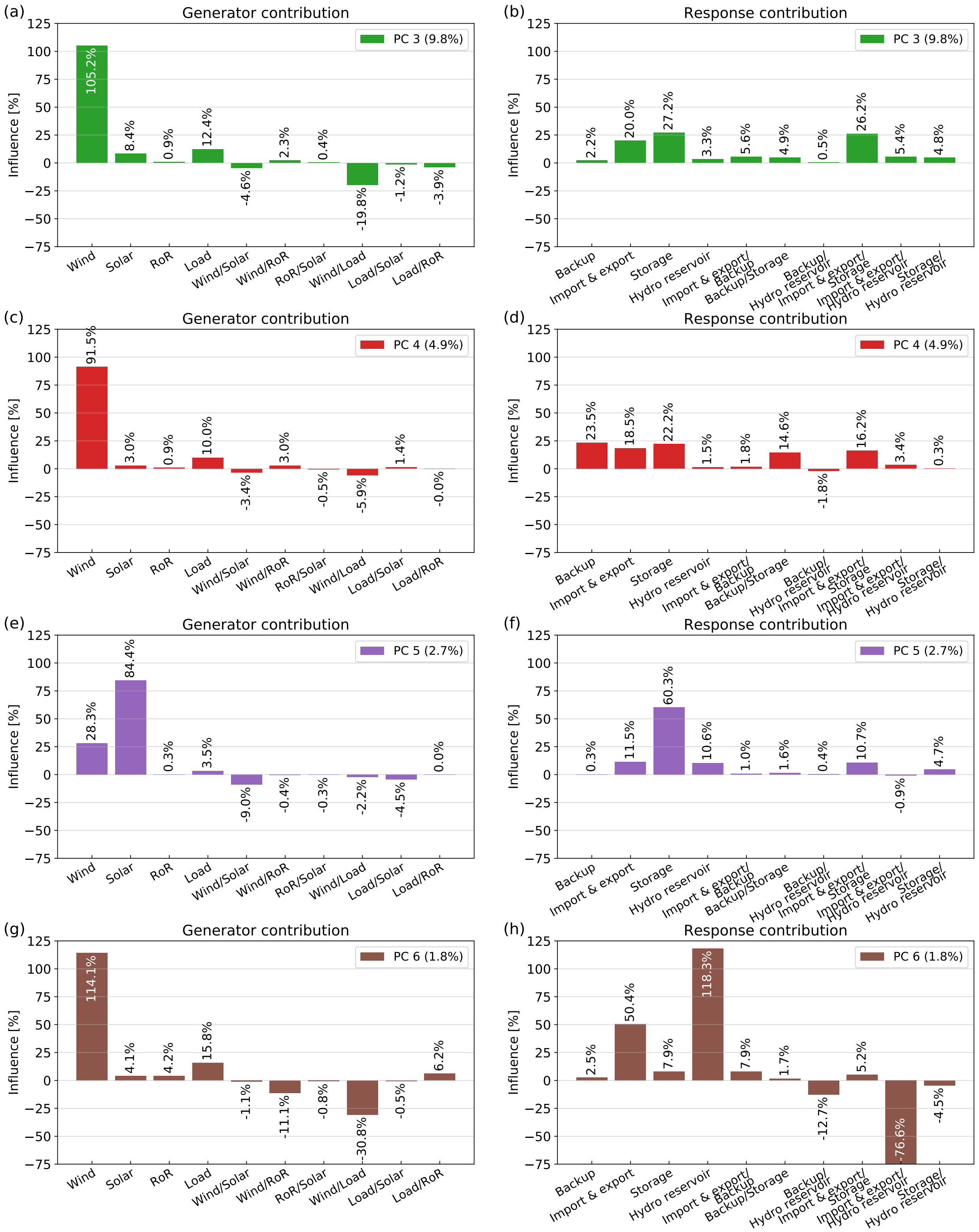

Figure S6: **Mismatch contribution characteristic overview for PCs 3 to 6, related to Figure 4**. For the mismatch PCs of the base case scenario we visualise the generator and response contributions for the PCs 3 (green), 4 (red), 5 (purple), and 6 (brown). Panels (a), (c), (e), and (g) visualise the generator and load contributions for the PCs 3 through 6, respectively. Similarly, the Panels (b), (d), (f), and (h) show the response contributions to the PCs 3 through 6, respectively. For the first two PCs see the two bottom panels in Figure 4 in the main text.

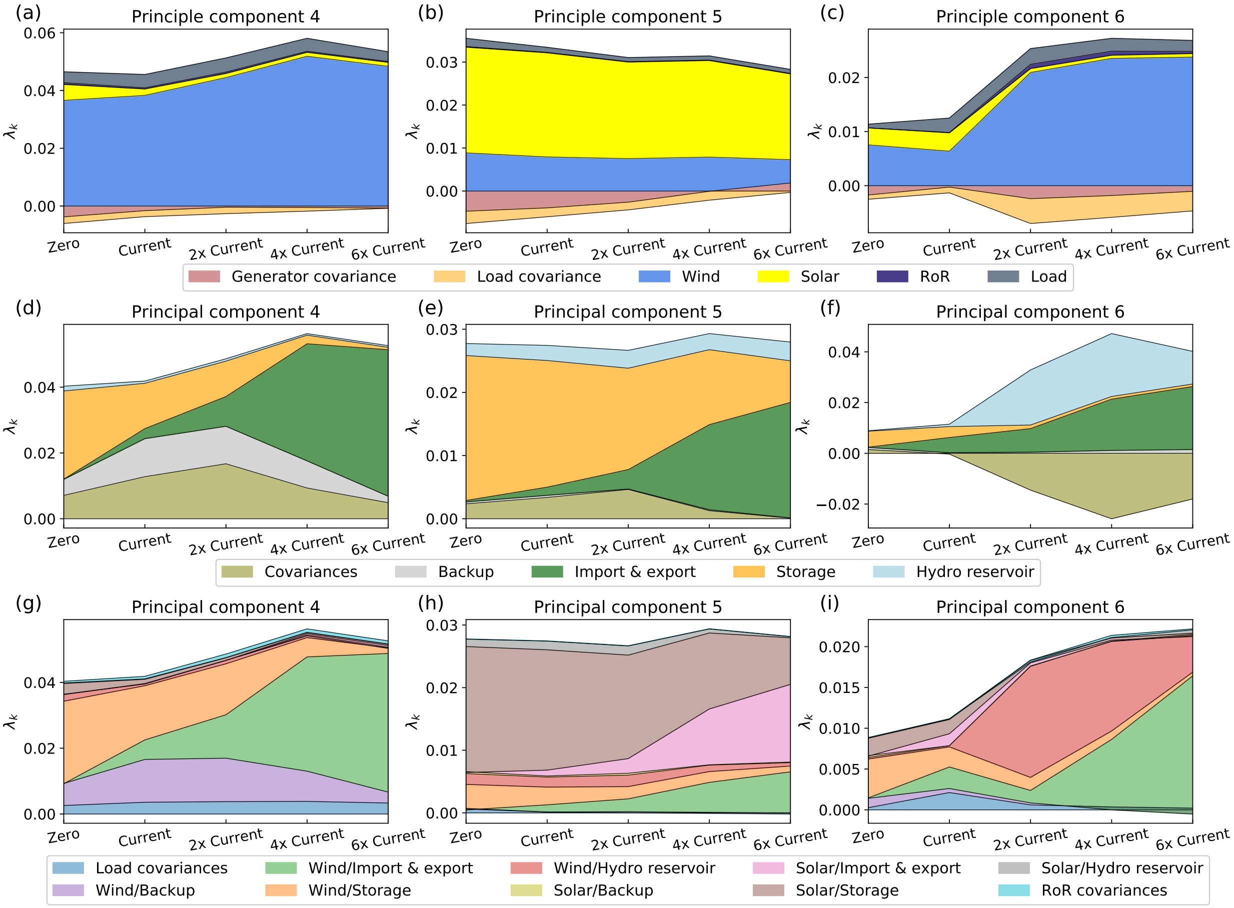

Figure S7: **Generator contributions and response contributions to the mismatch PCs four to six as a function of the transmission grid extension, related to Figure 8**. On the Panels (a), (b), and (c) we show the contributions of the variable renewable generators and the load together with their covariance terms for the respective PCs four, five, and six. Similarly, on the Panels (d), (e), and (f) we show the results of the system response contributions for the respective PCs four, five, and six. Note that the covariance terms can both be positive and negative and especially that the covariance term is negative in Panel (f) and subtracts from the top. A 95% CO_2 emission reduction compared to 1990 values is enforced for each of the transmission scenarios. Note that the base case scenario is represented by the centre scenario in each panel. See also Figure 8 in the main text for the results of the first three PCs.

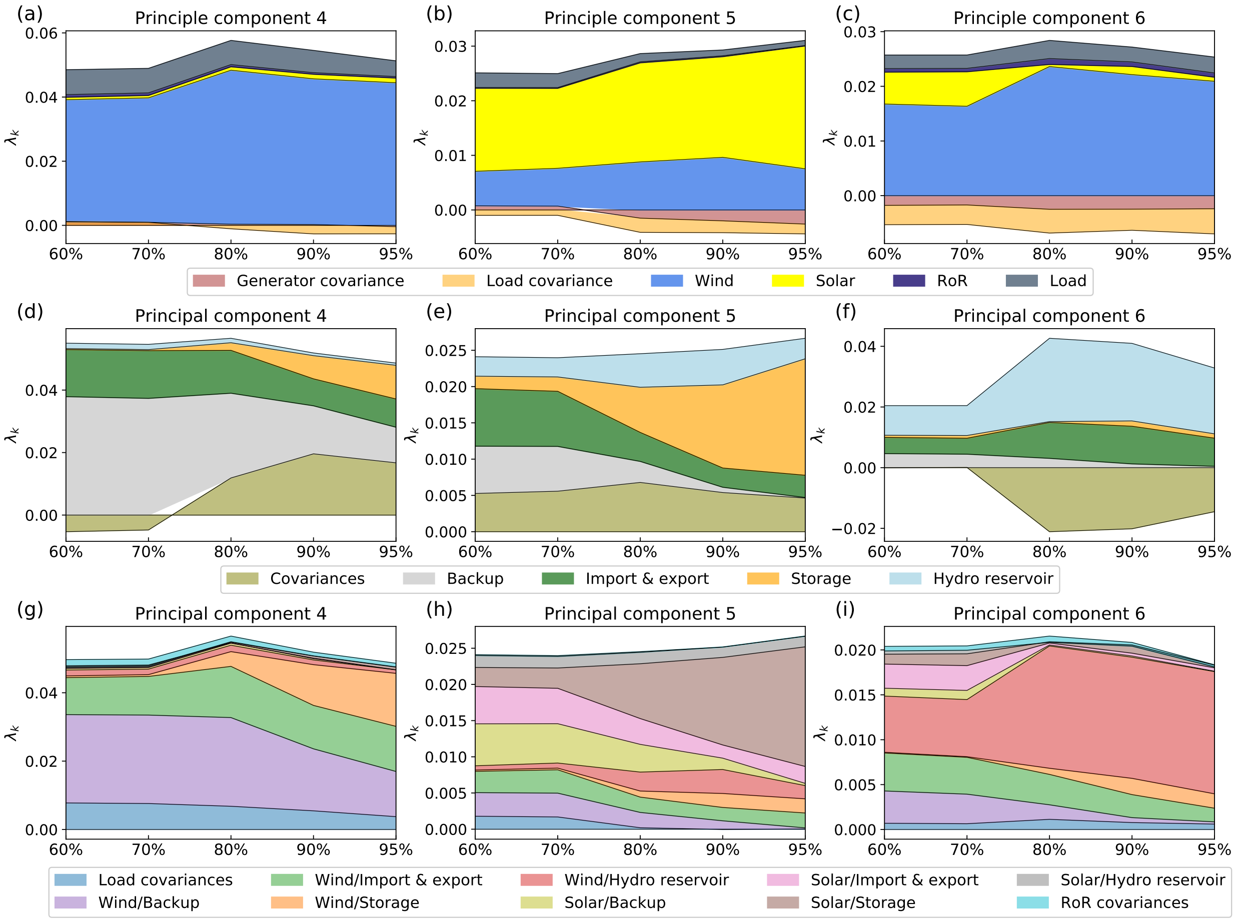

Figure S8: **Renewable generator and load contributions to the mismatch PCs, together with the corresponding response contributions, as a function of the total emission reduction relative to 1990-values, related to Figure 9**. The Panels (a), (b), and (c) show the generator and load contributions for the respective PCs four, five, and six. In the lower part of the figure, we show the response contributions to the mismatch PCs four, five, and six on the Panels (d), (e), and (f), respectively. Note that the covariance terms can both be positive and negative and that especially the covariance term is negative in Panel (f) and subtracts from the top. As in the base case scenario. the transmission grid is twice as strong as today's levels in each of the scenarios. Note that the base case scenario is represented by the rightmost scenario in each panel. See also Figure 9 in the main text for the analogous results of the first three PCs.
